# Use of intravenous lipid therapy in a cat with carprofen overdose

**DOI:** 10.1002/ccr3.2772

**Published:** 2020-02-29

**Authors:** Nathan S. Chumbler, Julie C. Schildt, Diane I. Mawby, Mark G. Papich

**Affiliations:** ^1^ University of Tennessee College of Veterinary Medicine Knoxville TN USA; ^2^ North Carolina State University College of Veterinary Medicine Raleigh NC USA

**Keywords:** feline toxicosis, intravenous lipids, NSAID overdose

## Abstract

Intravenous lipid emulsion (ILE) was administered to a cat with no adverse effects. This case report postulates that ILE can be used for the treatment of carprofen toxicity in cats and supports the lipid sink theory as the main mechanism of action.

## Graphical abstract

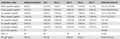

## 1 INTRODUCTION

A 12‐year‐old, male castrated domestic short hair ingested approximately 12 mg/kg of carprofen. He was treated with activated charcoal, intravenous lipid emulsion (ILE), and general supportive care. Serum concentrations measured pre‐ILE and 1, 3, and 6 hours post‐ILE treatment revealed increasing serum carprofen concentrations. The patient made a full recovery.

Nonsteroidal anti‐inflammatory (NSAID) toxicosis is one of the most common poisonings reported in small animals.^1^ Carprofen is a popular NSAID prescribed to dogs. Similar to other NSAIDS, it inhibits the production of cyclooxygenase which in turn decreases prostaglandin synthesis.^2^ This mechanism accounts for the drug's therapeutic effects of reducing pain and inflammation. However, reduction in prostaglandins can lead to adverse effects including gastric irritation, hepatic, and renal damage, and prolonged bleeding time by prevention of platelet aggregation.^2^

Elimination of carprofen occurs primarily by biotransformation into glucuronide metabolites within the liver.^3^ Cats have reduced glucuronidation enzyme activity decreasing their ability to metabolize carprofen. The elimination half‐life is approximately 18‐20 hours in the cat,^4^, ^5^, ^6^ whereas the half‐life in dogs is approximately 8‐12 hours.^4^, ^7^

Carprofen is approved for use in cats in Europe, Australia, and New Zealand at 4 mg/kg subcutaneously/intravenously as a single‐use perioperative analgesic. This dosing typically results in an 18‐ to 24‐hour duration of action when given intravenously or subcutaneously^5^; however, studies reviewing the dynamics and kinetics from oral administration in the cat are lacking.^2^ Toxicosis has been reported by the ASPCA Animal Poison Control Center at doses as low as 2.9 mg/kg in cats (Toxicology Brief – Volmer, P & Mensching D – Managing acute carprofen toxicosis in dogs and cats – 2009; http://veterinarymedicine.dvm360.com/toxicology-brief-managing-acute-carprofen-toxicosis-dogs-and-cats). One case report demonstrated duodenal perforation in a cat receiving carprofen at 2.2 mg/kg orally every 12 hours for an unreported duration of time.^8^ Although this patient also received dexamethasone and flunixin meglumine, they were administered after the onset of clinical signs; therefore, their administration was not thought to contribute to duodenal perforation. In contrast, a safety study reported no adverse effects in 7 healthy cats administered subcutaneous carprofen at 4 mg/kg once daily.^3^

Treatment of NSAID overdose typically consists of gastrointestinal decontamination (GID) using a combination of emesis and activated charcoal (AC).^9^, ^10^ These decontamination methods have been proven to cause a reduction in plasma carprofen concentration.^10^ Intravenous lipid emulsion (ILE) is also used for treating NSAID toxicosis in dogs. A dog with ibuprofen toxicosis^11^ and three dogs with naproxen toxicosis^12^ were treated successfully with ILE in combination with GID. Since carprofen has a similar mechanism of action and similar chemical properties as other NSAIDS, treatment of toxicosis with ILE should be equally successful. To the author's knowledge, this is the first report on the use of ILE in a cat with NSAID overdose.

## 2 CASE SUMMARY

A 12‐year‐old, 6.3 kg castrated male domestic short hair presented for increased vocalization while attempting to urinate and apparent urethral spasms; however, he urinated on the way to the hospital. Diagnostic testing including a serum biochemistry panel and abdominal/thoracic point‐of‐care ultrasound was unremarkable (Table 1). He was given a presumptive diagnosis of feline lower urinary tract disease and discharged with prazosin (Prazosin Hydrochloride 1 mg capsules, Mylan Pharmaceuticals Inc) (0.04 mg/kg PO q 8 hours) and buprenorphine (Buprenex^®^, Reckitt Benckiser Healthcare (UK) Ltd.) (0.02 mg/kg sublingually q 8 hours). At home the following morning, he inadvertently received a 75 mg carprofen (Rimadyl^®^) tablet (~12 mg/kg) and immediately returned for evaluation.

**Table 1:** Pertinent laboratory values at presentation, 24 h after carprofen ingestion and throughout hospitalization

Laboratory value	Initial presentation	24 h	Day 2	Day 3	Day 4	Day 5	Reference interval
BUN, mmol/L (mg/dL)	8.9(25)	12.5(35)	9.6(27)	10.4(29)	13.6(38)	11.1(31)	6.4‐14.3 (18‐40)
Creat, μmol/L (mg/dL)	133(1.5)	318(3.6)	212(2.4)	195(2.2)	195(2.2)	159(1.8)	79.6‐176.8 (0.9‐2.0)
Ca, mmol/L (mg/dL)	2.4(9.6)	2.0(8.1)	2.2(8.6)	2.2(8.7)	2.2(8.9)	2.2(8.7)	2.2‐2.7 (9.0‐10.8)
Phos, mmol/L (mg/dL)	1.16(3.6)	2.91(9.0)	1.26(3.9)	1.23(3.8)	1.58(4.9)	1.58(4.9)	0.7‐1.7 (2.2‐5.3)
Na, mmol/L (mEq/L)	156(156)	160(160)	156(156)	156(156)	149(149)	152(152)	145‐154
K, mmol/L (mg/dL)	3.6(3.6)	3.4(3.4)	3.3(3.3)	3.5(3.5)	3.0(3.0)	3.1(3.1)	2.5‐4.6
Cl, mmol/L (mEq/L)	117(117)	122(122)	116(116)	114(114)	111(111)	113(113)	114‐124
ALT, U/L (units/L)	77	133	‐	‐	‐	‐	29‐109
PCV, %	39	40	‐	30	30	‐	34‐48
TP, g/L (g/dL)	76(7.6)	78(7.8)	‐	64(6.4)	61(6.1)	‐	66‐84 (6.6‐8.4)

Abbreviations: ALT, alanine aminotransferase; BUN, blood urea nitrogen; Ca, calcium; Cl, chloride; Creat, creatinine; K, potassium; Na, sodium; PCV, packed cell volume; Phos, phosphorus; TP, total protein

On presentation, the patient was bright, alert, and responsive. Rectal temperature, heart rate, and respiratory rate were within normal limits. General physical examination was unremarkable, and a small bladder was appreciated on abdominal palpation. Given the elapsed time since ingestion was approximately 1 hour, initial therapy centered around GID. Emesis induction with hydromorphone (Hydromorphone HCl Injection, USP (2 mg/mL), West‐Ward,) (0.1 mg/kg IM) and dexmedetomidine (Dexdomitor^®^) (0.002 mg/kg IM) was unsuccessful. The cat was hospitalized for continued decontamination and supportive care which included a single dose of AC with sorbitol (Toxiban^®^ Suspension w/Sorbitol, Lloyd, Inc) (2 g/kg) administered via nasogastric tube (Mila Weighted Nasogastric Feeding Tube 5Fr X 55 cm, MILA International Inc). An IV catheter (20 g IV Catheter 1′′/25 mm) was placed, and the patient received Intralipid 20% (Intralipid^®^ 20%, Baxter Healthcare) (1.5 mL/kg over 15 minutes followed by 0.25 mL/kg/min for 1 hour). No adverse events were noted except gross lipemia. Blood was obtained for carprofen concentration measurement just prior to ILE administration then subsequently at 1, 3, and 6 hours post‐ILE. Serum was separated, frozen, and stored at −80°C for analysis by high‐pressure liquid chromatography (Agilent 1200 Series Gradient HPLC System, Agilent Technologies). Prior to the start of ILE, plasma carprofen concentration was 8 mcg/mL. Post‐ILE concentrations increased to 31.4 and 31.7 mcg/mL at 1 and 3 hours, respectively but decreased to 26.69 mcg/mL at 6 hours postadministration (Table 2).

**Table 2:** Serum carprofen concentrations measured by high‐performance liquid chromatography pre‐ and 1, 3, and 6 h post‐ILE

Time	Carprofen (mcg/mL)
Pre	8
1 h	31.4
3 h	31.7
6 h	26.69

Other therapy included Normosol‐R (Normosol‐R^®^, Hospira, Inc) (2 mL/kg/h), maropitant (Cerenia^®^) (1 mg/kg IV q 24 hours), pantoprazole (Protonix^®^IV, Premier Healthcare Alliance, LP, Wyeth Pharmaceuticals Inc) (1 mg/kg IV q 24 hours), and buprenorphine (Buprenex^®^, Reckitt Benckiser Healthcare (UK) Ltd.) (0.02 mcg/kg IV q 8 hours). During the first night of hospitalization, the patient began straining to urinate; therefore, a decompressive cystocentesis was performed. The following morning, an indwelling urinary catheter (Slippery Sam™ Tomcat Urethral Catheter 3.5Fr, Smiths Medical ASD INC) was placed due to continued straining and inability to urinate. Subsequent abdominal radiographs revealed a single urolith within the urinary bladder. Serum biochemical profile revealed a new azotemia, hyperphosphatemia, hypernatremia, and increased alanine aminotransferase activity (Table 1). Complete blood count was unremarkable except for a new mild anemia (Table 2). Over the next 3 days, he was treated with intravenous fluids, an indwelling urinary catheter, and continued administration of all medications. On the fourth day of hospitalization and stabilization, he underwent perineal urethrostomy and cystotomy. He recovered from surgery without incident and was discharged on Day 5.

## 3 DISCUSSION

Intravenous lipid emulsion is becoming increasingly popular in veterinary medicine as an adjunctive treatment of various intoxications including local anesthetics, baclofen, permethrins, and ivermectins.^13^, ^14^, ^15^ There are also reports of using ILE therapy in canine patients with NSAID overdose with reported positive outcomes. Given the large overdose in this case and risks associated with slow clearance in cats, ILE was chosen in addition to traditional GID to help reduce toxin absorption and prevent adverse effects. Although there have been reports of the use of ILE in feline intoxications, to the authors’ knowledge this is the first report of the use of ILE in feline NSAID overdose.

The prevailing theory for ILE therapy's mechanism of action is the “lipid sink” model, which postulates that the toxic compound is sequestered into a lipid compartment within the bloodstream.^16^ Essentially, lipid therapy expands the lipid compartment within the intravascular space which draws tissue bound drug off cellular receptor sites and into plasma, thereby trapping it in the transiently expanded lipid phase.^17^ The lipid sink theory was demonstrated in a study using a dye surrogate to visualize local anesthetic sequestration by lipid emulsion.^18^ More recently, the static “lipid sink” model has been refined into a dynamic “lipid shuttle/subway.” This description takes into account the redistribution of a xenobiotic molecule from target tissue and shuttling it to a less threatening location for storage, metabolism, and/or excretion.^19^ This theory was demonstrated in a pharmacokinetic study of bupivacaine overdose in rats, whereby plasma concentration of bupivacaine rapidly increased after ILE and subsequently rapidly declined due to shuttling to the liver for metabolism/excretion.^20^ Other potential mechanisms whereby ILE therapy may be beneficial for use in intoxications include (a) improving mitochondrial function by providing a source of fatty acids for metabolism, (b) providing cardiomyocytes with energy substrate, and (c) improving cardiomyocyte function by increasing intracellular calcium.^21^

The ability of the emulsion to sequester intoxicants is based upon the lipophilicity of the drug. This is expressed as its log P which is the partition coefficient measuring the solubility of a compound between 2 solvents.^16^ The higher the log *P* value, the more lipophilic the compound becomes. Drugs that are classified as lipophilic typically have a log *P* > 1.0.^16^ The log P of carprofen is 4.13 making it is highly lipophilic.^16^

This patient's serum carprofen concentration peaked after ILE administration. This result is supportive of the “lipid sink/shuttle” theory, because administration of the ILE likely sequestered the drug within the intravascular emulsion redistributing it from the tissues into the intravascular space. Measurement of parallel triglyceride and carprofen levels could further support this theory; it would be expected that as triglyceride levels decrease, the plasma concentration of carprofen should decrease. This theory has been documented in a case report where ILE therapy was used to treat lamotrigine and bupropion overdose in a 17‐year‐old girl.^17^ Serum bupropion levels paralleled triglyceride levels before and after lipid infusion, whereas no such increase and decrease in parallel with triglyceride levels was noted for lamotrigine. Bupropion is a highly lipophilic drug with a log P of 3.47 as compared to lamotrigine with a log P of −0.19.^17^

The ability of the lipid sink to redistribute pharmacologically active molecules was also described in a physiologically based pharmacokinetic model investigating local anesthetic overdose in humans. This study reported a reduction in concentration of bupivacaine at the tissue level and increases in total plasma concentrations in the presence of lipids; however, it also concluded that the lipid sink is not the sole mechanism by which IV administered lipid emulsion reverses local anesthetic systemic toxicity.^22^

The assay utilized in this report measures both the bound and unbound fraction of the drug. Therefore, the assay represented the total amount and does not distinguish between the free drug and the protein‐bound fraction. Despite the high protein binding of carprofen, eventual accumulation into the lipid matrix occurs due to the protein‐bound and unbound fraction existing in equilibrium. As the unbound fraction becomes taken‐up by the lipid matrix, the unbound fraction will be replaced to maintain this equilibrium.^23^ High concentrations of carprofen in plasma after treatment with lipids are likely due to the attraction to the lipids in plasma and a decreased distribution to the tissue compartment.

Dosing recommendations and indications for ILE administration in human patients are described in a position statement published by the American College of Medical Toxicology (ACMT).^24^ Recommended dosing regimen is a 1.5 mL/kg bolus over 2‐3 minutes followed by a continuous rate infusion at a rate of 0.25 mL/kg/min over 1 hour. In human patients, it is suggested that ILE doses should be limited to a maximum of 10% of the total blood volume to avoid lipid overload and limit possible complications arising from increased triglyceride concentrations.^25^ The dose chosen in this patient was similar to the recommended dose listed in the position statement from the ACMT on ILE. At the author's institution, repeat doses are given every 2‐6 hours based upon the persistence of clinical signs, as well as the presence of lipemia within the serum. Additional doses were not given to this cat based upon the absence of clinical signs, presence of lipemic serum, and the concern for possible adverse effects.

Most of the potential risks of ILE can be extrapolated from parenteral nutrition administration and include “fat overload syndrome” causing fat embolism, hemolysis, hyperlipidemia, and increased clotting times; volume overload; pancreatitis; nausea and vomiting.^26^ Specific to cats, two cases of corneal lipidosis have been reported following ILE administration for permethrin toxicosis.^27^, ^28^ Both of these cats received well over the recommended 10% of total blood volume dose, and the only side effect noted was corneal lipidosis in conjunction with persistent lipemia and hypertriglyceridemia. A recent study reviewing the effects of ILE on amitriptyline in the rat model postulated that an increased hemodynamic instability was noted in rats given ILE was due to enhancement of drug absorption from the GI tract.^29^ Further studies examining the pharmacokinetics and pharmacodynamics of ILE in veterinary patients are warranted to better understand appropriate dosing.

Surprisingly in this patient, the peak plasma concentration of carprofen was much lower than expected. The values listed within Table 2 are similar to or less than values that have been previously recorded when cats were administered 4 mg/kg intravenously at the same time intervals.^5^ It is possible the administration of activated charcoal prior to measuring carprofen levels and beginning ILE contributed to the reduced plasma carprofen concentrations. Several studies in dogs have shown that AC reduces plasma carprofen concentrations^10^, ^30^; however, the effectiveness of AC in reducing carprofen concentration has not been investigated in cats to the authors’ knowledge.

Unfortunately, this patient developed a urinary obstruction during hospitalization, so it is unknown if the development of azotemia was due to renal or postrenal causes. Given that the patient was receiving IV fluids throughout hospitalization and assessed daily for signs of dehydration, prerenal causes of azotemia were considered unlikely. A urinalysis to look for signs of possible acute kidney injury such as casts or protein was not performed which may have been useful in supporting the mechanism of azotemia. In the previous reports of ILE therapy for ibuprofen and naproxen overdose in dogs, only 1 of the 4 cases developed azotemia.

Future studies could prospectively evaluate the plasma concentrations of carprofen pre‐ and post‐ILE administration to further confirm that the lipid sink mechanism increases the plasma concentrations of the drug; thereby shuttling within the plasma for excretion. This could be monitored alongside triglyceride levels to prove that while triglyceride levels are high (due to ILE administration) plasma concentrations remain high. As the drug and lipid emulsion are excreted through the kidney, the triglyceride and drug plasma concentrations should decrease accordingly.

The ability of ILE therapy in preventing adverse effects associated with NSAID overdose in this case is unknown. The patient did not demonstrate any gastrointestinal side effects but did develop azotemia. Unfortunately, due to the development of a urethral obstruction, postrenal causes of azotemia cannot be excluded. Serum levels of carprofen did increase suggesting drug shuttling/sequestration in the plasma limiting exposure to target organs. However, this does not necessarily translate into clinical improvement as sufficient drug still may reach the target organ causing damage. Additionally, other decontamination methods may play a role in reducing complications associated with NSAID overdose. Future studies measuring carprofen concentrations after ILE therapy as compared to standard GID could help support the effectiveness of ILE therapy.

Although the patient's recovery cannot be attributed to one specific therapy, this report supports the lipid sink mechanism of action of ILE, whereby a toxin is sequestered in the intravascular space preventing it from causing damage to the target tissue. Intravenous lipid emulsion administration at the reported doses was well tolerated with no adverse effects other than transient lipemia. Given the reduced ability of feline patients to metabolize NSAIDs, ILE may play an important role in the management of toxicosis in this species.

## CONFLICT OF INTEREST

None declared.

## AUTHOR CONTRIBUTIONS

NSC: was the main author, conceived and designed the study, analyzed and interpreted the data, and drafted and critically revised the manuscript. JCS: was the main co‐author, conceived and designed the study, analyzed and interpreted the data, and drafted and critically revised the manuscript. DIM: was a co‐author, conceived and designed the study, analyzed and interpreted the data, and critically revised the manuscript. MGP: was a co‐author, analyzed and interpreted the data, and critically revised the manuscript.
